# Peer-led lifestyle interventions for the primary prevention of cardiovascular disease in community: a systematic review of randomised controlled trials

**DOI:** 10.1186/s12889-024-18328-w

**Published:** 2024-03-14

**Authors:** Geok Pei Lim, Jamuna Rani Appalasamy, Badariah Ahmad, Kia Fatt Quek, Amutha Ramadas

**Affiliations:** 1 Jeffrey Cheah School of Medicine and Health Sciences, Monash University Malaysia, Bandar Sunway, Selangor Malaysia; 2 School of Pharmacy, Monash University Malaysia, Bandar Sunway, Selangor Malaysia

**Keywords:** Cardiovascular disease, Peer-led, Peer support, Lifestyle intervention, Primary prevention, Community

## Abstract

**Background:**

Peer-led lifestyle interventions have gained recognition as effective approaches for managing and preventing chronic diseases. However, there remains a critical knowledge gap regarding the impact and effectiveness of peer-led interventions specifically in the primary prevention of cardiovascular disease (CVD). Our systematic review aims to synthesise the available evidence and evaluate the impact of peer-led lifestyle interventions, providing invaluable insights that can guide the development of peer-led strategies for preventing CVD.

**Methods:**

Systematic database searches were conducted on Ovid Medline, Embase, Cochrane Centre for Controlled Trials, PubMed and Scopus to source peer-reviewed articles published between 2013 and 2023. Reference lists of the included publications were also manually searched.

**Results:**

Fourteen unique randomised controlled trials were identified, of which three were pilot studies. Most of the interventions were conducted among individuals at moderate to high risk of CVD and lasted for a year. There is a variety of components in intervention delivery, including group discussions and individual counselling. Peer leader training mostly covered intervention delivery, communication, and research-specific skills. Systolic blood pressure showed the most promising CVD-related improvement, while mixed results were found for several other dietary and lifestyle behavioural outcomes.

**Conclusion:**

Peer-led lifestyle interventions have shown varying effectiveness in cardiovascular health outcomes. The competencies and roles of peer leaders were identified to guide future intervention development with a more comprehensive approach to the primary prevention of CVD.

**Supplementary Information:**

The online version contains supplementary material available at 10.1186/s12889-024-18328-w.

## Background

Cardiovascular disease (CVD) is a formidable force in global health, accounting for approximately 18 million deaths yearly [1]. The burden of CVD is increasing worldwide, with low- and middle-income nations experiencing a disproportionate impact [1]. Lifestyle risk factors, including unhealthy diet, physical inactivity, tobacco use, and alcohol consumption, contribute significantly to the growing prevalence of CVD [2]. Despite considerable health promotion efforts and advancement of CVD treatments, “lifestyle medicine” that could modify these risk factors remains the key to primarily preventing this disease, especially in high-risk populations [3, 4].

Lifestyle interventions that target adverse health behaviours have been shown to be potentially effective in reducing CVD risk, and guidelines on lifestyle management have been made available by the American Heart Association [5]. However, the translational strategies of lifestyle interventions into practice may not be more scalable in community settings due to a lack of workforce for implementation, cost considerations, program acceptability, and fidelity within the local context [6]. Furthermore, healthcare professionals may lack the requisite skills, time, socio-cultural experiences, and empathy for providing on-going support and empowerment in behavioural change [7]. As such, the importance of social environment and support for closing the gap in pragmatic behaviour change interventions in the community cannot be overstated [8].

Peer support is essential in promoting and maintaining various complex health behaviours, preventing, and managing various non-communicable diseases (NCDs) [9]. This is because peers, who share similar backgrounds and experiences, offer a sense of community and ongoing emotional support, thus could reinforce motivation to overcome barriers in sustaining behaviour change [10]. In view of the functional role of peer support in the public health system, peer-led lifestyle interventions have emerged as a promising approach to promoting healthy behaviours in community settings. For instance, the success of peer-led interventions has been demonstrated in several health promotion programs, including weight loss [11], physical activity improvement [12] and smoking cessation [13].

As for the prevention and management of NCDs, several systematic reviews reported favourable effects of peer-led interventions on diabetes [14], cancer [15] and mental health [16]. The reviews also extended to the management and secondary/tertiary prevention of CVD [17]. With the growing number of trials attempted for peer-led interventions in the primary prevention of CVD, there still needs to be synthesised evidence on their effectiveness. Hence, our study aimed to evaluate the effectiveness of peer-led lifestyle interventions for the primary prevention of CVD in community settings. The review also examined the characteristics of successful peer-led interventions, including the training provided to peer leaders, roles of peer leaders, and other support.

## Methods

### Study design

This systematic review was conducted following the Preferred Reporting Items for Systematic Reviews and Meta-Analyses (PRISMA) 2020 Statement [18] and checklist (Supplementary Table S1). The review protocol has been registered with the International Prospective Register of Systematic Reviews (PROSPERO) (Registration ID: CRD42023415838) and can be accessed publicly through https://www.crd.york.ac.uk/prospero/display_record.php?RecordID=415838 [assessed on 29 April 2023].

### Search strategy

We conducted an extensive literature search to identify relevant studies in five electronic databases: Ovid Medline, Embase, Cochrane Centre for Controlled Trials, PubMed, and Scopus. The search strategy was devised using the keywords and Boolean operators (‘cardiovascular disease’ OR ‘heart disease’) AND (‘peer support’ OR ‘peer educator’ OR ‘peer group’) AND (lifestyle).

Our searches were limited to randomised controlled trials (RCTs), English language, and articles published from 2013 to 2023. This timeframe was selected to ensure that the evidence obtained was current. The complete search syntax and the strategy for the electronic databases are shown in Supplementary Table S2. We also manually searched relevant studies by examining the reference lists and citations of the included studies and previous reviews.

### Study selection

The study selection process was conducted systematically using the Covidence software [19]. All searched records obtained from the databases were imported into Covidence, and duplicates were removed automatically. The articles were first screened by their titles and abstracts, followed by the screening of full-text articles based on the eligibility criteria. Conflicts were resolved through discussion and consensus between the two reviewers (GPL & AR). Studies that did not meet the criteria were excluded by consensus.

We included all RCTs (including pilot RCTs) that reported on the effectiveness of lifestyle interventions facilitated by peers or community members in changing health-related behaviours and/or health outcomes related to CVD in community settings. Only studies involving the adult population (older than 18 years old) were included. We excluded reviews, non-randomized interventions, conference abstracts, book chapters, monographs, dissertations, grey literature, and study protocols. Interventions conducted in clinical settings without peer-based components or any report on health outcomes were also excluded.

### Quality assessment

The methodological quality of the included studies was assessed using the Cochrane Collaboration’s tool for assessing the risk of bias in randomised trials [20]. This assessment tool consists of and provides information on six domains of bias, including (i) selection bias (random sequence generation and allocation concealment), (ii) performance bias, (iii) detection bias, (iv) attrition bias, (v) reporting bias, and (vi) other bias. Two reviewers (GPL and AR) assessed each domain of individual study, and all included studies, which were then classified as “low risk”, “high risk” and “unclear risk” of bias. Any disagreements were resolved through discussion between the two reviewers (GPL and AR).

### Data extraction and synthesis

We extracted the relevant information of the eligible studies using a Google Sheet-based template. The data extracted include year, country, age of participants, sample size, study duration, intervention and control regimens, peer-based components, measured outcomes, and primary findings. Subsequently, the characteristics and main findings of the included studies were qualitatively synthesised using the narrative approach. We did not perform a meta-analysis in this review due to heterogeneity in the intervention design and outcome data.

## Results

### Study selection

**Fig. 1:**
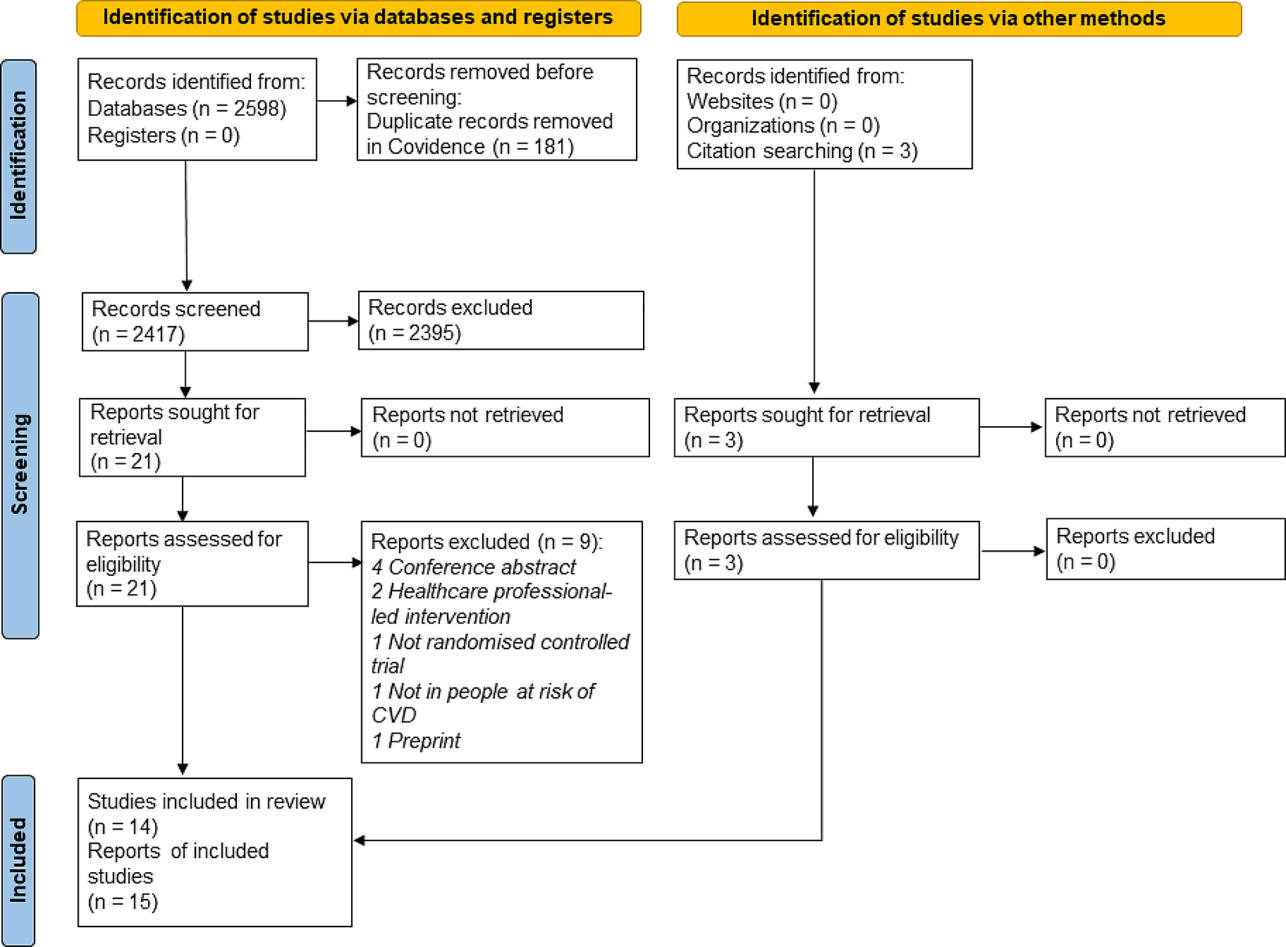
PRISMA 2020 flow chart showing the study selection process

Figure 1 presents the PRISMA flow chart illustrating the study selection process. The systematic database search yielded a total of 2598 records. After eliminating duplicates, 2417 titles, and abstracts were screened. Next, 21 full texts were sought for retrieval and then assessed based on the eligibility criteria. Most reports were excluded due to conference abstract (n = 4) and two were interventions delivered by healthcare professionals. A manual search of the included papers’ reference lists and citations was done and yielded three additional records. Subsequently, 15 articles representing 14 unique studies that matched the predefined criteria were finalised for this review. The details of included studies were summarised in Supplementary Table S3.

### Study characteristics

The general characteristics of the included studies are summarised and presented in Table 1. Of the 14 unique RCT studies, three were pilot RCTs. The sample size for pilot RCTs ranged from 31 to 114 individuals, while the RCTs ranged between 223 and 3539 individuals. With regards to the study origins, there were five studies conducted in America [22, 25, 31, 34–35], five in South Asia [26–30], followed by four studies in the Europe region, whereby three were in the United Kingdom [21, 32, 33] and one in Spain [23, 24]. Five studies targeted low-income populations [21–22, 25, 27, 34], and two studies focused on rural regions [28, 30]. The pilot study by O’Neill et al. (2022) focused on established community groups instead of individuals, including the peer support group and minimum support group [33]. Most of the studies (6/14) were conducted among participants at moderate to high risk of CVD as specified using a risk score [28, 32] or having at least two risk factors [26, 31, 34, 35]. Except for a study by Wijesuriya et al. [26], that included participants both under and above 18 years old, the mean age of participants ranged between 42 and 63.5 for all the included studies. The recruitment sites were scattered throughout various community locations [22–23, 25–26, 30–33, 35] as well as households [28–29]. Participants were also recruited using practice lists [21], voter lists [27], and administrative data [34]. Most studies (9/14) had study evaluation up to at least a year, while there was a study using end-point evaluation with median of three years of follow-up [26].

**Table 1:** General characteristics of included studies (n = 9)

Study (Year)	Country	Study population	Sample size	Mean ± SD age (Years)	Recruitment site	Study evaluation
*Goodall et al. (2014) [21]	United Kingdom	Adults from deprived communities at risk of CVD	114	Intervention group = 53.7 ± 12.5 Control group = 52.6 ± 14.2	Recruitment letter to eligible adults on practice list	Baseline & 6-month follow-up
Koniak-Griffin et al. (2015) [22]	United States	Low-income, overweight, immigrant Latino women	223	44.6 ± 7.9	Parent education centres, churches, laundromats, and organisations providing basic services to children and families.	Baseline, 6- & 9-month follow-ups
Gómez-Pardo et al. (2016) [23]; Fernández-Alvira et al. (2021) [24]	Spain	Adults at risk of CVD	543	42 ± 6	Multicentre in 7 municipalities	Screening, baseline, 1-year & 2-year follow-ups
He et al. (2017) [25]	Argentina	Low-income adults with uncontrolled hypertension	1432	Intervention group = 56.1 ± 13.6 Control group = 55.5 ± 13.0	Primary health care centres	Baseline, 6-month, 12-month & 18-month follow-ups
Wijesuriya et al. (2017) [26]	Sri Lanka	Urban healthy participants at high risk of CVD	3539 (1814 aged above 18 years old)	Mean (range) Intervention group = 22.5 (6–40) Control group = 22.4 (7–40)	National Diabetes Centre	Baseline & end-point evaluation with median 3 years of follow-up
Neupane et al. (2018) [27]	Nepal	Adults in a low-income population	1638	*Normotension* Intervention group = 42.17 ± 9.79 Control group = 42.25 ± 9.52 *Prehypertension* Intervention group = 46.02 ± 9.73 Control group = 45.15 ± 9.92 *Hypertension* Intervention group = 50.12 ± 8.99 Control group = 50.28 ± 8.14	Community-based survey using voter list	Baseline & 1 year follow-up
Joshi et al. (2019) [28]	India	Adults from rural households with intermediate to high risk of CVD	2312 households (3261 individuals)	Intervention group = 61.7 ± 10.23 Control group = 61.7 ± 10.38	Rural households	Baseline, 12-month & 18-month follow-ups
Khetan et al. (2019) [29]	India	Adults with CVD risk factor(s)	1242	Intervention group = 52.1 ± 9.6 Control group = 51.7 ± 9.8	Homes	Baseline & 2-year follow-up
Gamage et al. (2020) [30]	India	Adults from rural regions with hypertension	1734	Intervention group = 56.6 ± 14.3 Control group = 56.9 ± 13.7	Community-based survey	Baseline & 5-month follow- up
Latina et al. (2020) [31]	Grenada	Adults from a small, middle-income country at high risk of CVD	402	51.4 ± 14.5	Parishes	Baseline, 6-month & 12-month follow-ups
*McEvoy et al. (2021) [32]	Northern Ireland	Non-Mediterranean population at high risk of CVD	75	57.1 ± 6.7	Advertisements at multiple locations	Baseline, 3-, 6- & 12-month follow-ups
*O’Neill et al. (2022) [33]	Northern Ireland	Established community groups with members at increased CVD risk	4 groups (31 participants)	Peer support group = 54.6 ± 8.7 Minimal support group = 63.5 ± 12.1	Community organisations	Baseline, 3-, 6- & 12-month follow-ups
Nelson et al. (2023) [34]	United States	Low-income veterans with multiple CVD risks	264	Intervention group = 60.3 ± 9.7 Control group = 60.9 ± 9.8	Administrative data from Veterans Health Administration primary care	Baseline & 12-month follow-up
Shah et al. (2023) [35]	United States	South Asians immigrants with T2DM and comorbid hypertension	190	Mean (95% CI) Intervention group = 56.2 (53.7, 58.7) Control group = 55.7 (53.4, 57.9)	Clinics and community-based referral	Baseline & 6-month follow-up

*=Pilot RCTs; SD = Standard deviation; T2DM = Type 2 diabetes mellitus

### Study quality assessment

**Fig. 2:**
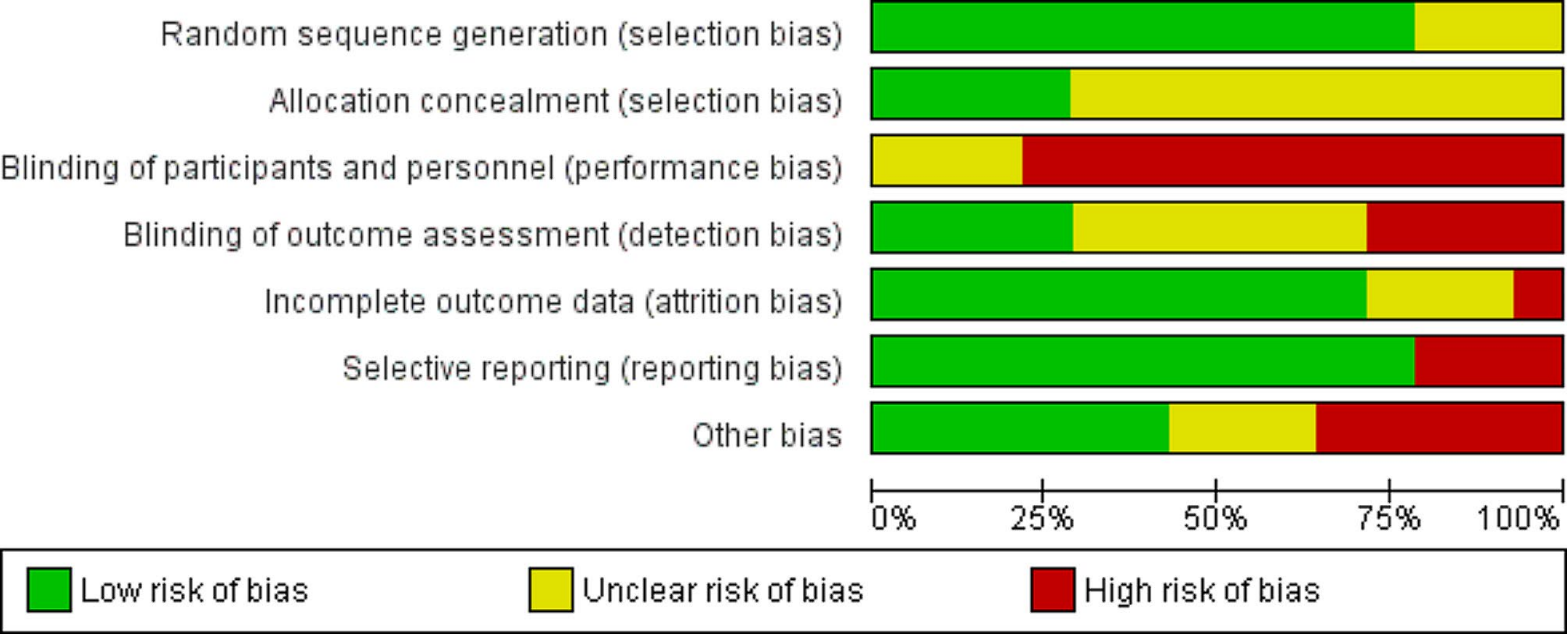
Risk of bias summary of included studies (n = 14)

All the included trials were of acceptable quality as shown in Fig. 2. However, a high risk of performance bias was detected for most of the studies. This is due to the administration of lifestyle interventions that precluded blinding of participants and personnel. As for selection and detection biases, most studies did not address the allocation concealment and blinding of outcome assessment, leaving the risk substantially unclear. The risk of attrition bias in the included studies was considered low due to the inclusion of intention-to-treat analysis in five studies [21–23, 31–32] and multiple imputation in three studies [25, 30, 34]. Besides, the reason for missing data was addressed as not related to outcome measures in one study [28]. While there was no serious issue pertaining to reporting bias, selective reporting was present in three studies. For instance, certain outcomes were stated in the trial registry or measured during data collection, but these were not reported in the study [26, 29, 33]. As for the risk of other bias, it was considered high in five studies due to a lack of sample size justification [21–22, 33–34] and study with an unequal number of subjects at baseline [27].

### Intervention characteristics

As we included lifestyle interventions facilitated by peers or community members, the intervention providers came in various designations, namely lay health trainers [21], community health workers and volunteers [22, 25, 27–30, 35], peer educators [23, 26], peer leaders [31–32], peer supporters [33], peer health coaches [34]. Collectively, we named these intervention providers as peer leaders thereafter. Most of the interventions (71%) lasted for a year [23, 25–29, 31–34]. Meanwhile, the shortest intervention duration was three months [21, 30] and the longest was the study using end-point evaluation with a median three years of follow-up [26]. In half of the included studies, the control groups received usual care lifestyle advice and/or written health information [21, 25–27, 29–30, 34]. Meanwhile, an educational program was provided to the control group participants in 5/14 of the studies [22, 24, 31–33]. All the included studies involved peer leader training prior to intervention and the various roles of peer leaders are described below.

#### Peer leader training

The duration of peer leader training varied from three hours to four weeks. The components of the training are presented in Table 2. The skills to deliver interventional modules and health information were the most important components in all studies, followed by communication skills (8/14) and research-specific skills (8/14). Research-specific skills included survey methods, measuring, recording, reporting, and following up [22, 25, 27–30, 34–35]. Five studies highlighted the emphasis on motivation skills, wherein peer leaders were trained in motivational techniques [34–35] and equipped to motivate participants for long-term behaviour change through the establishment of short-term goals [21, 24, 30]. Besides that, peer leaders were supported with motivational sessions by psychologists [23] and monthly training refresher sessions [26]. Leadership skills were trained in four studies [23, 31–33], group facilitation skills were emphasised in three studies [30, 32–33], while subject engagement skills were provided in two studies [21, 35].

**Table 2:** Components of peer leader training (n = 14)

Study	Duration	Delivery of modules & health promotion	Communication skills	Research-specific skills	Motivational skills	Leadership skills	Group facilitation skills	Engagement skills
Goodall et al. [21]	10 days	√	√		√			√
Koniak-Griffin et al. [22]	100 h	√		√				
Gómez-Pardo et al. [23]; Fernández-Alvira et al. [24]	6 h	√	√		√	√		
He et al. [25]	2 days	√		√				
Wijesuriya et al. [26]	4 weeks	√	√					
Neupane et al. [27]	5 days	√		√				
Joshi et al. [28]	4 weeks	√		√				
Khetan et al. [29]	7 days	√	√	√				
Gamage et al. [30]	5 days	√		√	√		√	
Latina et al. [31]	3 h	√	√			√		
McEvoy et al. [32]	14 h	√	√			√	√	
O’Neill et al. [33]	14 h	√	√			√	√	
Nelson et al. [34]	100 h	√		√	√			
Shah et al. [35]	3 days	√	√	√	√			√

#### Peer leader roles

The roles of peer leaders of each intervention are depicted in Table 3. Most peer leaders had at least two roles when delivering the interventions. There were seven studies conducted individual meetings [21, 25–29, 34] while group meetings were conducted in six studies [23, 30–33, 35]. Intervention by Koniak-Griffin et al. was the only study that involved group education followed by individual teaching and coaching [22].

**Table 3:** Roles of peer leaders (n = 9)

Study	Frequency	Advice on diet & lifestyle behaviour	Discussion & reflection	Dynamic activities	Monitor progress	Teaching & coaching	Assess readiness to change	Goal setting
Goodall et al. [21]	Monthly	√					√	√
Koniak-Griffin et al. [22]	Monthly					√^a^		
Gómez-Pardo et al. [23]; Fernández-Alvira et al. [24]	Monthly		√	√	√			√
He at al. [25]	Weekly	√			√	√^b^		√
Wijesuriya et al. [26]	Trimonthly	√					√	
Neupane et al. [27]	Quarterly	√			√			
Joshi et al. [28]	Bimonthly	√			√			
Khetan et al. [29]	Bimonthly	√			√			
Gamage et al. [30]	Fortnightly				√	√^c^		
Latina et al. [31]	Monthly		√					
McEvoy et al. [32]	Monthly		√	√	√			√
O’Neill et al. [33]	Monthly		√	√				√
Nelson et al. [34]	Monthly				√	√^b^		√
Shah et al. [35]	Monthly				√	√^c^		√

^a^ 8-weekly group education was provided by peer leaders prior to individual teaching and coaching.

^b^ Individual coaching.

^c^ Group education.

The individual meetings were done via home visits plus phone calls or text messaging, ranging from a weekly to quarterly frequency. Among the seven studies that included individual sessions, all the peer leaders provided advice on healthy diet and lifestyle behaviours [21, 25–29] while health coaching modules were completed in one study [34]. Five studies included progress monitoring by measuring blood pressure [25, 27–29, 34] and readiness to change was assessed in another two studies [21, 26].

In studies involving group sessions (n = 6), all meetings were held monthly, with each session lasting between one to two hours [23, 31–33, 35], except for the study by Gamage et al. [30], which conducted education and monitoring fortnightly. During the meetings, the peer leaders were responsible for delivering education and facilitating discussion, reflection and experience sharing on healthy dietary and lifestyle behaviours to reduce CVD risk among group members. Challenges and improvements for behavioural changes were also discussed. Two interventions had the core educational content to promote adoption and adherence to the Mediterranean diet [32–33]; thus, practical food demonstrations were conducted during the meeting sessions. Peer leaders also organised other dynamic activities, including menu design, sporting activities and relaxation techniques [23]. Four studies involved progress monitoring and feedback by peer leaders [23, 30, 32, 35], while goal setting was carried out at each group meeting in four studies [23, 32–33, 35].

#### Other support/ resources

In addition to meetings with peer leaders, several other forms of support and resources were provided to the intervention group participants. For instance, participants received a health handbook containing information on CVD prevention, and it was used to record lifestyle behaviour, health parameters and immediate goals [23]. In studies promoting the Mediterranean diet, both control and intervention groups received written educational materials. However, only participants in peer support groups were given a personal workbook to facilitate dietary goal setting and self-monitoring of personal dietary goals [32–33]. To promote preventive therapies, participating households were provided short goal-directed slogans printed on common household objects [28]. Additionally, to facilitate blood pressure monitoring, participants in the intervention were provided with blood pressure monitors in three studies [25, 34, 35].

### Outcomes

**Fig. 3:**
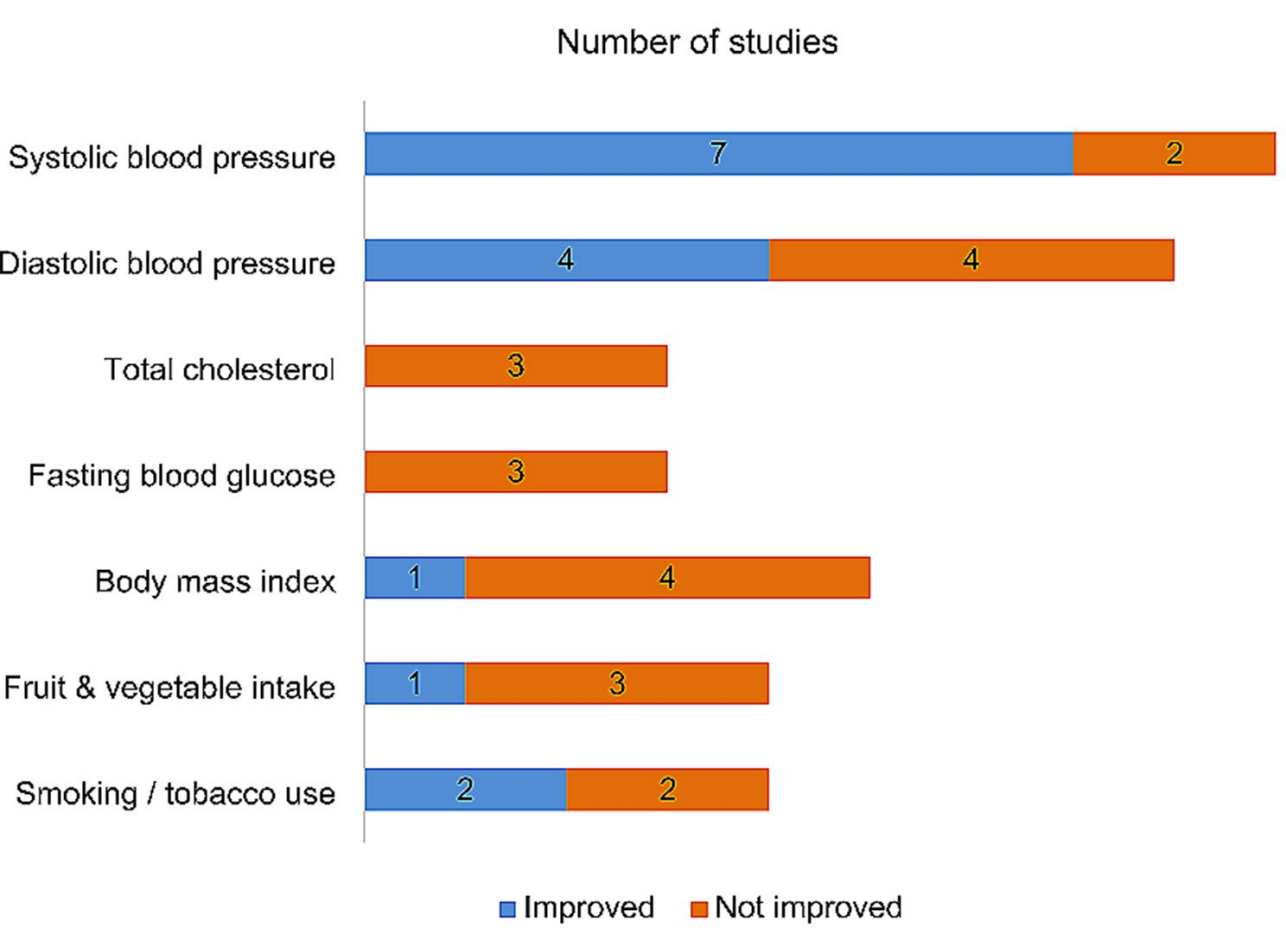
CVD-related outcomes of the included studies (n = 13)

All study outcomes reported in the included studies are presented in Supplementary Table S4. Due to the variability of the study aims and intervention designs, the extracted outcome measures are broadly classified into clinical outcomes, dietary and lifestyle behaviour outcomes, and other outcomes for comparisons. Figure 3 summarises the CVD-related outcome measures of the included studies. One pilot study without hypothesis testing [33] was excluded from the outcome comparison.

#### Clinical outcomes

Systolic blood pressure was the most common clinical outcome reported in 9/14 studies, out of which seven showed improvements post-intervention [25, 27–30, 32, 35]. Nonetheless, only five interventions showed significant differences between groups at follow-up [25, 27, 29–30, 35]. While four studies showed significant changes in diastolic blood pressure over time, the changes were similar between intervention groups in study by McEvoy et al. [32]. In the other studies, significant differences were observed between control and intervention groups [25, 30, 35]. Total cholesterol, high-density lipoprotein cholesterol, low-density lipoprotein cholesterol and triglycerides were not showing improvements in all four studies that assessed these outcomes [21–22, 32, 34].

Fasting blood glucose was assessed in three studies, with no significant changes observed post-intervention [22, 29, 32]. Glycated haemoglobin (HbA1c) levels were assessed in only two studies [32, 35], and improvements were observed across intervention groups (minimal support, peer support and dietitian support groups) in study by McEvoy et al. [32]. Out of five studies, only one showed improvement in body mass index (BMI) [32]. Waist circumference was significantly decreased in one intervention [22], and another intervention notably reduced the cardio-metabolic endpoints, including new onset hypertension and dysglycemia [26].

#### Dietary outcomes

Six studies assessed the dietary outcomes relating to cardiovascular health using various parameters. The fruit and vegetable intake were improved from baseline in only one of the four studies [21, 27, 30, 35] that assessed this outcome, but the changes were not significantly different from the control group [21]. The intervention by Koniak-Griffin and colleagues significantly improved heart-healthy dietary habits among the interventional subjects [22]. The Mediterranean diet scores, as in the study by McEvoy et al. were improved across the intervention groups [32]. Intervention by Gamage et al. demonstrated greater reduction in added salt intake and alcohol consumption than in the usual care group [30]. Meanwhile, study by Shah et al. significantly showed reduction in sugar sweetened beverage intake in both intervention and control groups [35].

#### Lifestyle behavioural outcomes

The study by Shah et al. [35] was the only intervention that significantly increased self-reported physical activity levels out of five studies that assessed this outcome. Nonetheless, the step counts measured in the study by Koniak-Griffin et al. were significantly improved at the 9-month follow-up, and the changes were more significant than in the control group [22]. Two out of four studies reported improvements in smoking and non-smoked tobacco use habits [28, 30] but only intervention by Gamage et al. demonstrated significant change between intervention and control groups [30].

#### Other outcomes

The Fuster-BEWAT score (FBS), which is a health metric assessing the CVD modifiable risk factors (blood pressure, exercise, weight, alimentation, tobacco) was measured in two interventions [23, 24]. Gómez-Pardo et al. reported that, at 1-year follow-up, the intervention group showed significantly higher overall FBS levels, and a greater increase compared to the control group [23]. However, the 2-year follow-up showed no notable differences between the groups in terms of the average FBS or changes in FBS post-intervention [24]. Another 1-year intervention study also did not show improvements in FBS [31]. Three studies assessed health-related quality of life [21, 31, 34], but only study by Nelson et al. [34] showed significant improvement in mental component summary in intervention group. The INTERHEART risk score declined significantly across the studied households [28]. Two out of three interventions improved adherence to antihypertensive drugs more significantly than control group [28, 35]. One study reported significant improvements in heart disease knowledge post-intervention [22].

## Discussion

Peer-led lifestyle interventions have emerged as a more scalable approach to preventing and managing various chronic diseases in the community [9]. This systematic review synthesised recent findings regarding the designs and effectiveness of peer-led lifestyle interventions in reducing CVD risk. We identified a total of 14 unique studies, of which eight were conducted among the low-and-middle-income populations [22, 25–28, 30–31, 34], while three pilot RCTs were conducted in the UK [21, 32–33]. This shows a focus on using peer educators to address health disparities among low- and middle-income populations. Nonetheless, pilot trials testing the feasibility and preliminary outcomes also noted a growing interest in peer-led interventions for CVD risk reduction in high-income regions.

The included interventions mainly targeted middle-aged individuals, particularly those exhibiting moderate to high risk of CVD underscores the critical aspect of preventive healthcare and healthy aging. Exceptionally, one study [26] included at-risk participants between 6 and 40 years old, highlighting the potential benefits of early intervention and prevention strategies. The various recruitment sites had also shown researchers’ proactiveness in reaching out to community members of various backgrounds and contexts, thus, increasing the representativeness and generalisability of the findings. Most studies had follow-up evaluations of at least a year representing the importance of assessing the sustainability and fidelity of the interventions in a longer term.

While lay health trainers and community health workers do not necessarily share the experiential knowledge of the health condition, they share common characteristics and functions that align with the peer leadership concept [36]. Based on their shared features of personal connection to the community, the provision of support and guidance, and the acquisition of specialised training, we acknowledged their role as peer leaders within the community. We also ensured consistency in discussing these intervention providers throughout the review. However, study quality varied and the heterogeneity precluded meta-analyses.

Notably, the vast variation in control group designs and follow-up periods has made the intervention effectiveness incomparable between studies. For instance, we found that the intervention that lasted for six months, with a control group receiving a completely different group education content, showed significant improvements in outcomes of the intervention group at a 9-month follow-up [22]. Meanwhile, two interventions that supplied ample educational materials to the control groups at baseline had rendered no significant intervention impacts as improvements were observed across the study groups [31–32]. Immediate evaluation post-interventions have shown significant improvements in health outcomes [23, 25, 27–30, 35]. However, post-follow-up evaluations after two years revealed no between-group difference in the mean FBS or its change from baseline [24]. This suggested the non-sustainable impacts of the interventions during the maintenance phase. In contrast, the study with endpoint evaluation of a median three years of follow-up [26] demonstrated the need for continuous peer support and lifestyle advice for preventing disease onset. Further research is needed to explore the optimal duration of interventions and the potential benefits of extended follow-up periods for sustained behaviour change.

For peer leader training, the competencies required to deliver interventional modules and health information were consistently recognized as the most important components. This highlights the critical role of peer leaders in effectively disseminating information and implementing intervention strategies. Communication skills were the second most essential skill, as these were trained in all five studies that facilitated group discussion [23, 31–33, 35] and three studies with individual advising [21, 26, 29]. Research-specific skills, including survey methods, measuring, recording, reporting and follow-up were also a common feature in peer leader training as provided in eight studies [22, 25, 27–30, 34–35].

Due to the heterogeneity of study objectives and intervention contents, diverse health outcomes related to cardiovascular health were assessed in different interventions. The FBS, which calculated the composite score for clinical (blood pressure and weight), dietary (fruits and vegetable intakes), and lifestyle behavioural (exercise and smoking) outcomes, was reported in two studies instead of individual parameters [23, 31]. Thus, the results of these outcomes could not be compared directly with other included studies. Both studies demonstrated improved FBS at the 1-year follow-up [23, 31]. However, the changes reported by Gómez-Pardo et al. were not sustained at the 2-year follow-up [24]. This suggests that the effects of the intervention may diminish over time. While the FBS was higher in the intervention group, no between-group differences were reported by Latina et al. as the improvements were also supplied in the control group due to the baseline educational session [31].

When comparing the intervention effects on blood pressure, either home visit or group education with blood pressure monitoring had shown to be beneficial and effective as compared to usual care [25, 27, 29–30, 35]. The reasons for non-significant differences in blood pressure between control and intervention groups in another four studies were attributable to normal baseline readings [22], adherence to antihypertensive treatment [28], ample health information provided across the study groups [32], and employment of telephone visits instead of home visits during the pandemic [34]. Meanwhile, the lack of improvements in blood lipid profiles could be due to the short duration (three and six months) [21, 22] and low intensity (dietary or modules-focused) of the interventions [32, 34]. However, the daily steps and waist circumference were improved in the 6-months intervention by Koniak et al., showing a behaviour change [22] that could potentially improve the lipid profile in the longer term [37]. There were no significant changes in fasting blood glucose [22, 32] as the baseline readings of this parameter were within the normal range. Nonetheless, the pilot trial by McEvoy et al. demonstrated marked dietary behaviour change was associated with improvements in BMI and HbA1c [32]. Finally, the long-term, trimonthly lifestyle advice intervention that significantly reduced the new onset hypertension and dysglycemia [26] underscored the importance of on-going peer support in the modification of behavioural risk factors.

As for the dietary outcomes, we found mixed effectiveness of peer-led lifestyle interventions in influencing specific dietary changes. Significant improvements in dietary habits [22], as well as reductions in salt and alcohol intakes [30], were observed in the intervention group, where group education was provided more frequently (weekly or fortnightly) and followed up in the later time point (two to three months after intervention). However, when group education was given monthly and followed up immediately post-intervention, the improvement in dietary outcomes did not differ from the control group [32, 35]. Our investigation indicated that more frequent education sessions may allow for greater reinforcement of key messages and behaviours, while follow-up at a later point may afford participants more time to implement and sustain dietary changes.

Regarding the lifestyle behavioural outcomes, the physical activity levels which were subjectively reported did not improve in most intervention as the participants were already undertaking moderate to high levels of physical activity at baseline [21, 23, 27, 30]. Meanwhile, the study using an objective measure of step counts recorded significantly favourable changes in physical activity [22]. This highlights the importance of using objective measures to assess physical activity outcomes accurately. Therefore, future interventions should consider incorporating objective measures to provide a more comprehensive understanding of the effect on physical activity behaviours. The group education provided fortnightly by Gamage et al. [30] was the only intervention that improved smoking habits significantly from the control group apart from bimonthly to quarterly home visits [27–29]. This could be explained by the group education approach that capitalises on the power of social influence, shared learning, and collective support contributing to an environment conducive to positive habit changes [38].

In addition, only one study assessed the participants’ knowledge of heart disease and showed improvement post-intervention [22]. In fact, knowledge assessment should be undertaken in lifestyle interventions as it serves as a foundation for empowering individuals to make informed decisions about their cardiovascular health. When participants have a solid understanding of the disease and its risk factors, they are more likely to engage in behaviour change, adhere to recommended guidelines, and take proactive steps toward preventing or managing cardiovascular conditions [39]. By addressing knowledge gaps and promoting accurate understanding, interventions can have a more meaningful and long-lasting effect on individuals’ cardiovascular health outcomes.

This systematic review has several strengths. Firstly, the review protocol was registered with PROSPERO. This helps to minimise bias and provides a record of the planned methods and objectives before conducting the review. Next, our literature search was comprehensive by including multiple electronic databases. We also manually searched reference lists and citations of included studies and previous reviews, enhancing the likelihood of identifying relevant studies. Thirdly, our review specified study selection within the last ten years to ensure the inclusion of current evidence. Besides, our review included interventions that consisted of interactive sessions led by community members to promote the inclusivity of studies in community settings. Lastly, the characteristics and main findings of included studies were qualitatively synthesised using a narrative approach, which is appropriate considering the heterogeneity of intervention design and outcome data.

Meanwhile, there were also limitations to note in this review. Firstly, only studies published in English were included, which may introduce language bias and potentially exclude relevant studies published in other languages. Secondly, the review did not perform a meta-analysis due to intervention design and outcome data heterogeneity. While this decision is justified, it limits the ability to provide a quantitative summary of the overall effect size. Finally, the present review primarily focuses on RCTs and may limit the inclusiveness and diversity of the evidence of other study designs considered in the review.

### Implications for practice

Our findings highlight the gaps exist in the current practices, particularly in the standardisation of training programs and the assessment of peer leader performance. It is imperative to establish standardised peer leader training programs focusing on core competencies such as knowledge of the subject matters, effective communication skills and research specific skills, as well as assessment tools and monitoring mechanisms to ensure the effectiveness and quality of peer-led interventions.

Besides, the current review underscores the importance of optimising intervention duration and follow-up schedules for enhancing the effectiveness of peer-led lifestyle intervention in reducing CVD risk. Prioritising more frequent education sessions and later follow-up evaluations can reinforce key messages and behaviour changes. Utilising objective measures and leveraging group education approaches can further enhance the intervention impact. Moreover, integrating knowledge assessment empowers individuals to make informed decisions about cardiovascular health.

Moving forward, it is essential for practitioners and policymakers to prioritize the development of standardised training frameworks, rigorous competency assessments, and robust performance monitoring systems. By addressing these challenges and implementing evidence-based strategies, we can optimise the effectiveness of peer-led interventions in promoting cardiovascular health and reducing CVD risk in communities.

## Conclusion

Peer-led lifestyle interventions have shown promise in preventing and managing cardiovascular disease risk factors, particularly among middle-aged individuals with moderate to high CVD risk. These interventions have effectively improved cardiovascular health outcomes with varying degrees of success. Group education conducted more than a fortnightly frequency together with blood pressure monitoring showed superior influence on clinical, dietary and lifestyle behavioural outcomes. By considering the competencies and roles of peer leaders, group-based interventions can be standardised with progress monitoring to improve lifestyle behaviours and reducing the burden of cardiovascular disease in the target population.

### Electronic supplementary material

Below is the link to the electronic supplementary material.

Supplementary Material 1

Supplementary Material 2

Supplementary Material 3

Supplementary Material 4

## Data Availability

All data generated or analysed during this study are included in this published article and its supplementary information files.
