# Supplementary material for: Peer-led lifestyle interventions for the primary prevention of cardiovascular disease in community: a systematic review of randomised controlled trials
Source: BMC Public Health. 2024 Mar 14;24:812. doi: 10.1186/s12889-024-18328-w (PMC10941612; doi:10.1186/s12889-024-18328-w)
Supplement: Supplementary file 1 — Supplementary Material 1 [file 12889_2024_18328_MOESM1_ESM.docx]

**Supplementary Table S4.** Outcomes reported by the included studies (n=13).

|  | | **Improved** | **Total** | **References** |
| --- | --- | --- | --- | --- |
| **Clinical outcomes** | Systolic blood pressure | 7 | 9 | [22,25,27-30,32,34-35] |
|  | Diastolic blood pressure | 4 | 8 | [22,25,27,29-30,32,34-35] |
|  | Total cholesterol | 0 | 3 | [21-22,32] |
|  | HDL-c | 0 | 2 | [22,32] |
|  | LDL-c | 0 | 2 | [22,34] |
|  | Triglycerides | 0 | 2 | [22,32] |
|  | Fasting blood glucose | 0 | 3 | [22,29,32] |
|  | HbA1c | 1 | 2 | [32,35] |
|  | OGTT | 0 | 1 | [32] |
|  | Body mass index | 1 | 5 | [22,28,30,32,34] |
|  | Body weight | 1 | 2 | [22,35] |
|  | Waist circumference | 1 | 1 | [22] |
|  | Waist hip ratio | 0 | 2 | [28,30] |
|  | Primary composite cardio-metabolic endpoint | 1 | 1 | [26] |
|  | Plasma Vitamin C | 1 | 1 | [32] |
|  | Serum lutein | 0 | 1 | [32] |
|  | Serum carotenoid | 0 | 1 | [32] |
|  | Plasma fatty acids | 0 | 1 | [32] |
| **Dietary outcomes** | Fruits & vegetables intake | 1 | 4 | [21,27,30,35] |
|  | Heart-healthy dietary habits | 1 | 1 | [22] |
|  | MDS | 1 | 1 | [32] |
|  | Salt intake | 1 | 2 | [27,30] |
|  | Alcohol intake | 1 | 3 | [21,27,30] |
|  | Sugar sweetened beverage intake | 1 | 1 | [35] |
| **Lifestyle behaviours** | Physical activity level | 1 | 5 | [21,23,27,30,35] |
|  | Steps | 1 | 1 | [22] |
|  | Smoking/ tobacco use | 2 | 4 | [27-30] |
| **Other outcomes** | Fuster-BEWAT score | 1 | 2 | [23,31] |
|  | Quality of life | 1 | 3 | [21,31,34] |
|  | INTERHEART risk score | 1 | 1 | [28] |
|  | Knowledge of heart disease | 1 | 1 | [22] |
|  | Framingham risk score | 0 | 1 | [34] |
|  | Drug adherence | 2 | 3 | [28,30,35] |

HbA1c= Glycated haemoglobin; HDL-c= High-density lipoprotein cholesterol; Questionnaire; LDL-c= Low-density lipoprotein cholesterol; MDS= Mediterranean diet score; OGTT= Oral glucose tolerance test
