# Supplementary material for: Peer-led lifestyle interventions for the primary prevention of cardiovascular disease in community: a systematic review of randomised controlled trials
Source: BMC Public Health. 2024 Mar 14;24:812. doi: 10.1186/s12889-024-18328-w (PMC10941612; doi:10.1186/s12889-024-18328-w)
Supplement: Supplementary file 2 — Supplementary Material 2 [file 12889_2024_18328_MOESM2_ESM.docx]

**Supplementary Table S3**. Summary of all included studies (n = 9).

| Study/ Country [Ref] | Objectives | Participants | Mean age ± SD (years) | Intervention provider | Intervention provider training | Intervention duration | CVD risk indicator | Control group | Intervention group | Setting | Data collection | Outcome measures | Main findings |
| --- | --- | --- | --- | --- | --- | --- | --- | --- | --- | --- | --- | --- | --- |
| *Goodall et al. (2014)  United Kingdom  [21] | To investigate the effectiveness of LHTs at promoting heart-healthy lifestyles. | 114 adults from deprived communities at risk of CVD | Intervention group  = 53.7 ± 12.5  Control group  = 52.6 ± 14.2 | Lay health trainers (LHTs) | 10-day training course focused on communication, client engagement and motivating long-term  behaviour change by establishing short-term goals, and enabling  the person to build self-efficacy. | 3 months | At least one CVD risk factor (self-reported):   - hypertension - raised total cholesterol - diabetes - obesity - currently smoking. | Received health information leaflets alone. | - Received LHT support plus health information leaflets. - LHTs assessed readiness to change, set goals, provided information and advice aimed at changing key beliefs, diet and lifestyle behaviours related to CVD - LHTs provided ongoing and effective helping relationship via phone/face to face over 3 months (3 – 6 sessions in total) | Phone/face to face | Baseline & 6-month follow-up | - Primary outcome: daily dietary intake of fruits & vegetables. - Secondary outcomes: health status (EQ-5D), physical activity, alcohol usage, and total cholesterol. | - Both groups had increased fruits & vegetables consumption at follow-up. - No significant changes in health status (EQ-5D), physical activity, alcohol usage, and total cholesterol. |
| Koniak-Griffin et al. (2015)  United States  [22] | To evaluate the effects of a lifestyle behaviour intervention delivered by specially trained promotoras to low-income, overweight, immigrant Latinas residing in Southern California. | 223 low-income, overweight, immigrant Latino women | Total  = 44.6 ± 7.9  Intervention group  = 43.3 ± 7.4  Control group  = 45.9 ± 8.2 | Community health workers (promotoras) | 100 hours of  structured training activities, focusing on  delivery of modules and research-specific skill sessions. | 6 months | Overweight | Received 6-month safety/disaster preparedness educational program. | - 8 weekly group education (2-h) classes based upon Your Heart, Your Life (Su Corazo´n, Su Vida) - Individual Teaching and Coaching to reinforce class content, assist achieving personal goals, support behaviour change, and provide guidance on how to overcome barriers to lifestyle behaviour change (4 home visits plus 4 telephone calls delivered over 4 months). | Community settings such as school classrooms. | Baseline, 6- and 9-month follow-ups | - Dietary habits - Physical activity (steps) - BMI - Weight - WC - SBP, DBP - LDL-c, HDL-c, TC, TG - FBG - Knowledge of heart disease | - Significant improvements in dietary habits, daily steps, WC, and CVD knowledge in the intervention group. |
| Gómez-Pardo et al. (2016) [23]; Fernández-Alvira et al. (2021) [24]  Spain | To analyse the effectiveness  of a peer support strategy in modifying the  behaviour of healthy individuals at risk of CVD. | 543 adults at risk of CVD | Participants at 1-year follow-up  = 42.0 ± 6.0  Participants at 2-year follow-up  = 48.0 ± 5.4 | Peer educators | - 3-h session on relevant health and health promotion information, leadership, and communication skills. - 3-h motivational session led by the psychologist by midterm. | 12 months | At least one of the risk factors:   - hypertension - overweight or obese - smoking - physical inactivity. | Attended 6 workshops on core lifestyle & risk factor education only. | - Attended 6 workshops on core lifestyle & risk factor education. - Monthly peer group meeting (60-90 mins) for reflections, sharing and dynamic activities. - Given a health handbook with information on prevention, and to record lifestyle behaviour, health parameters & immediate goals.   *Theory：Social cognitive theory* | Not specified | Initial (screening), post-workshops  (randomization / baseline), final assessments (1-year  follow-up), and follow-up assessments (2 years). | - Fuster-BEWAT Score (FBS) (BP, exercise, weight, alimentation, and tobacco) - Anthropometric measures (BP, weight, height & WC) - Health behaviours (physical activity, smoking, adherence to Mediterranean diet, QOL) | - At 1-year follow-up, the intervention group showed a significantly larger overall FBS and greater increase in FBS compared with the control group. - At 2-year follow-up, no between group differences in FBS mean and FBS changes, except the weight component improved more in the intervention group. |
| He et al. (2017)  [25]  Argentina | To test whether a CHW–led multicomponent intervention would improve blood pressure (BP) control among low-income patients with uncontrolled hypertension. | 1432 low-income adults with uncontrolled hypertension | Intervention group  = 56.1 ± 13.6  Control group  = 55.5 ± 13.0 | Community health workers (CHWs) | - 2-day training on lifestyle modification, home BP-monitoring. | 18 months | Uncontrolled  BP (systolic ≥140mmHg, diastolic ≥90mmHg, or both) | Received usual care pertaining to current practices for  hypertension management at the community level. | - CHW-led home-based intervention (health coaching and home BP-monitoring and audit) - Given an automatic home BP monitor and log. - Weekly individualised text messaging to improve lifestyle changes and medication adherence. | Home | Baseline, 6-month, 12-month & 18-month follow-ups | - Primary outcomes: SBP, DBP - Secondary outcomes: Proportion   of patients who had controlled hypertension, adherence to  antihypertensive medication, treatment intensification | - Greater reductions in SBP and DBP in the intervention group. - The proportion of patients with controlled hypertension increased more in the intervention group. |
| Wijesuriya et al. (2017)  Sri Lanka  [26] | To compare a trimonthly pragmatic  LSM (P-LSM) programme with a less-intensive 12  monthly LSM (C-LSM) programme on a primary composite  endpoint of predictors of cardio-metabolic disease in young urban healthy participants. | 3539 urban healthy participants at high risk of CVD | Mean (range)  Intervention group  = 22.5 (6-40)  Control group  = 22.4 (7-40) | Peer educators | - 4-week training by specialist nutritionists & diabetes specialists - Monthly training refresher sessions | The median (range)  follow-up was 3 (1-4) years. | At least 2 risk factors:   - First degree (parental) family history of T2DM - Physical inactivity - Raised BMI - Raised WC | Received lifestyle advice annually. | Received lifestyle advice every 3 months, on a one-to-one basis. | Not specified | Baseline & end-point evaluation with median 3 years of follow-up. | Primary composite cardio-metabolic endpoint was new onset T2DM, hypertension, IGT, IFG, CVD and renal disease. | - After a median follow-up of 3 years, the cumulative incidence of the primary composite cardio-metabolic endpoint was n = 479 in P-LSM (74 per 1000 person years) versus 561 in C-LSM (96 per 1000 person years), with an IRR of 0.89 (95% CI 0.83–0.96, P = 0.002). - New onset hypertension & dysglycemia significantly reduced with P-LSM. |
| Neupane et al. (2018)  Nepal  [27] | To assess the effectiveness of an FCHV-led  lifestyle intervention and screening of BP in reducing BP in individuals who are normotensive,  prehypertensive, or hypertensive in Nepal. | 1638 adults in a low-income population | **Normotension**  Intervention  = 42.17 ± 9.79  Control  = 42.25 ± 9.52  **Prehypertension**  Intervention  = 46.02 ± 9.73  Control  = 45.15 ± 9.92  **Hypertension**  Intervention  = 50.12 ± 8.99  Control  = 50.28 ± 8.14 | Female community health volunteers (FCHV) | 5-day intensive training course on BP. | 1 year | Not specified | Received usual care pertaining to current practices for  hypertension management at the community level. | Home visits by FCHVs every 4 months for lifestyle counselling and BP monitoring. | Home | Baseline & 1 year follow-up | - Primary outcome: mean systolic blood pressure (SBP) at 1 year. - Secondary outcomes: change in mean diastolic blood pressure (DBP) and percentage change in proportion of risk factors (smoking, alcohol consumption, high salt intake, and low physical activity). | - The mean SBP at 1 year was significantly lower in the intervention group than in the control group for all cohorts. - The differences in change in DBP were only significant for participants with hypertension. |
| Joshi et al. (2019)  India  [28] | To assess impact of CHW-based interventions in reducing CVD risk factors in rural households in India. | 2312 rural households  (3261 participants) with intermediate to high risk of CVD | Intervention group  = 61.7 ± 10.23  Control group  = 61.7 ± 10.38 | CHWs | 4-week trainings on  survey methods, measurement  techniques and lifestyle  advice to modify CVD risk factors. | 12 months | INTERHEART risk score:   - >10% = moderate risk - >20% = high risk | No CHW visits | CHWs visited intervention households every 2 months for measuring BP, ascertaining, and reinforcing adherence to prescribed therapies, providing short goal- directed slogans printed on common household objects. | Home | Baseline, 12-month & 18-month follow-ups | - Primary outcomes: SBP and adherence to prescribed BP-lowering drugs. - Secondary outcomes: INTERHEART risk score, BMI, WHR, tobacco use. | - Significant decline in SBP from baseline in both groups, no between group differences. - Adherence to antihypertensive drugs was greater in intervention households. - Proportion of individuals who use smokeless tobacco significantly declined between baseline and 12 months in the intervention arm. |
| Khetan et al. (2019)  India  [29] | To assess the effectiveness of the use of CHWs to manage hypertension,  diabetes, and smoking in an integrated manner in improving control of these risk factors. | 1242 adults with CVD risk factor(s) | Intervention group  = 52.1 ± 9.6  Control group  = 51.7 ± 9.8 | CHWs | 7-day, 3-h training on intervention delivery and data collection. | 2 years | At least one risk factor:   - Hypertension - Diabetes - Smoking | Received a handout at the end of the screening process, that explained their respective risk factor and brief verbal advice from the field worker. | - Received bimonthly home visits and lifestyle counselling by CHWs. | Home | Baseline & 2-year follow-up | - Primary outcome: change in SBP/ FBG/ daily cigarettes/ bidis smoked - Secondary outcomes: mean reduction in DBP, control rates of hypertension and diabetes, proportion of participants with diabetes who were on a statin or aspirin, proportion of participants with hypertension and tobacco use on a statin, proportion of tobacco users who quit smoking, and mean reduction in weight and WC, respectively. | The intervention led to improved systolic blood pressure in hypertension, an inconclusive effect on FBG in diabetes, and no demonstrable effect on smoking. |
| Gamage et al. (2020)  India  [30] | To determine the effectiveness of a CHW-led group-based education and monitoring program for the management of hypertension. | 1734 adults from rural regions with hypertension | Intervention group  = 56.6 ± 14.3  Control group  = 56.9 ± 13.7 | CHW | 5-day course on delivering community-based program. | 3 months | Hypertension | Received usual care pertaining to current practices for  hypertension management at the community level. | 6-fortnightly sessions of BP monitoring, education about hypertension and its management, and support for lifestyle changes. | Community setting in the villages. | Baseline & 5-month follow- up | - Primary outcome: proportion of people with controlled hypertension - Secondary outcomes: SBP, DBP, use of antihypertensive medication, BMI, waist-hip ratio, lifestyle behaviours | - Control of BP improved from baseline to follow-up more in the intervention group. - Greater decline in SBP and DBP in the intervention group. |
| Latina et al. (2020)  Grenada  [31] | To study the effects of peer education groups on their CV risk factors, quality of life, and health-related behaviours. | 402 adults at high risk of CVD | 51.4 ± 14.5 | Peer leaders | 3-hour training session on leadership  and communication skills in addition to the relevant  healthy behaviour promotion. | 12 months | At least 2 CV risk factors:   - elevated BP - overweight or obese - high WC - elevated blood glucose level - low level of physical activity - low fruit and vegetable intake - dyslipidaemia - currently smoking. | Received the series of educational lectures at the time of enrolment, followed by self-management for 1 year. | Monthly peer group meeting to discuss educational lecture topics. | Local parish | Baseline, 6-month & 12-month follow-ups | - Primary outcome: Fuster-BEWAT score (FBS) (BP, anthropometric measurements, IPAQ, lifestyle assessment) - Secondary outcome: quality of life | - At post intervention, the mean FBS was higher in the intervention group compared to the control group. - No significant improvement in FBS after peer-group intervention for 1 year. - No between-group difference in the change of FBS. |
| *McEvoy et al. (2021)  Northern Ireland  [32] | 1. To test recruitment strategies and estimate retention/attrition  rates.  2. To estimate and compare the variability of Mediterranean Diet Score (MDS) over the 12-month study between the peer support intervention and other intervention groups.  3. To estimate and compare the variability of biochemical markers of nutritional status and health markers over the course  of the intervention, as for MDS, between the peer support  intervention and other intervention groups.  4. To estimate the sample size for a large-scale trial. | 75 non-Mediterranean population at  high risk of CVD | Total = 57.1 ± 6.7  Peer support group (PSG) = 55.7 ± 6.6  Minimal support group (MSG) = 58.0 ± 5.1  Dietitian support group (DSG) = 57.8 ± 8.1 | Peer leaders | 2-day training (14h) delivered by qualified experts in dietary behaviour change and group facilitation skills. | 12 months | Joint British Societies CVD risk prediction charts score ≥20 % | MSG received a written Mediterranean Diet (MD) information booklet at baseline. | **PSG:**   - Attended 2-h monthly group sessions (n=11) by peer leaders, consisting of MD and/or behavioural education components for discussion, and practical food demonstrations. - Given MD information booklet and personal workbook to facilitate dietary goal-setting and self-monitoring of personal dietary goals.   **DSG:**   - Individual face-to-face 90 min motivational interview with a study dietitian at baseline. - Provided with key MD foods over the 12-month intervention for daily consumption. - 2-h quarterly structured group education session led by the dietitian + 15 min individual progress review and feedback. | Convenient location within the community setting | Baseline, 3-, 6- & 12-month | - MDS - Nutritional biomarkers as indicators of MD adherence (plasma vitamin C, fatty acids, serum carotenoids, lutein) - Health markers, including CVD risk factors (body weight, BP) - Markers of diabetes risk (blood glucose and glycated haemoglobin (HbA1c) levels). | - Significant increase in MDS in all groups over 12 months. - Significant difference over time between groups was apparent in analysis with the augmented MDS and a reduction in MDS by 12 months was suggestive of poorer maintenance in the PSG group. - No intergroup difference in change in CVD & T2DM risk factors over the study period. |
| *O’Neill et al. (2022)  Northern Ireland  [33] | To evaluate the feasibility of a peer support intervention to encourage the adoption and maintenance of a Mediterranean diet (MD)  in established community groups where existing social support may assist the behaviour change process. | Four established community groups  with members at increased CVD risk  (31 participants) | PSG = 54.6 ± 8.7  MSG = 63.5 ± 12.1 | Peer supporters | 2-day training (14h) delivered by qualified experts in dietary behaviour change and group facilitation skills plus post-session phone calls. | 12 months | At least one CVD risk factor:   - T2DM and NOT on any medication - Currently smoking - Hypertension - On anti-hypertensive medication - Elevated LDL-c - Low HDL-c - On cholesterol-lowering medication - Overweight/ obese - Family history of premature coronary heart disease (CHD) - Ethnicity (South Asian (Afghanistan, India, Pakistan, Bangladesh, Sri Lanka, Nepal, Bhutan, and Maldives); or African Caribbean). | MSG received written educational literature MD diet at baseline visit. | - PSG attended 2-h monthly group sessions (n=11) by peer leaders, consisting of MD and/or behavioural education components for discussion, practical demonstrations, and set goals. - Provided with a range of MD educational resources. - Given personal workbook to facilitate dietary goal-setting and self-monitoring of personal dietary goals. | Usual community group venue | Baseline, 3-, 6- and 12-month follow-ups | - Primary outcome: change in MDS at 6 months from baseline (adoption) - Secondary outcome: change in MDS at 12 months from 6 months (maintenance) - Others: weight, BMI, WC, HC, BP | Non-inferential data:   - A > 2-point increase in MDS was observed in both the PSG and the MSG at 6 months, maintained at 12 months. - An increase in MD adherence was evident in both groups during follow-up. - Trends towards improvement in CVD risk factors were observed in the PSG group only. |
| Nelson et al. (2023)  United States  [34] | To test the effectiveness of a home-visit, peer health coaching  intervention to improve health outcomes for veterans with multiple CVD risks. | 264 low-income veterans with multiple CVD risks | Total  = 60.6 ± 9.7  Intervention group  = 60.3 ± 9.7  Control group  = 60.9 ± 9.8 | Peer health coaches | 100 h of comprehensive training in health coaching and motivational interviewing. | 12 months | - Diagnosed hypertension - At least one BP measurement ≥150/90 mmHg documented in the past 12 months - One other self-reported CVD risk (current smoking, overweight or obesity, and/or diagnosis of hyperlipidaemia) | Received usual medical care and educational materials. | - Received 5 home visits and 5 telephone visits by peer health coaches over 12 months. - Received a manual of educational materials, an automatic BP monitor, a scale, a pill organizer, and healthy nutrition tools. | Home | Baseline & 12-month follow-up | - Primary outcome: SBP - Secondary outcome: percentage of respondents meeting prespecified normal BP goal (≤120/80mmHg), DBP, health-related QOL, CVD risk, prior year health care use | - No difference was found in change in SBP between the intervention and control groups. - Intervention group reported greater improvements in mental health-related QOL scores. |
| Shah et al. (2023)  United States  [35] | To test the feasibility and  efficacy of a CHW-led, patient-centred lifestyle telehealth  intervention to improve BP control and T2DM management  among South Asian adults. | 190 South Asians immigrants with T2DM and comorbid hypertension | Mean (95% CI)  Intervention group  = 56.2 (53.7, 58.7)  Control group  = 55.7 (53.4, 57.9) | CHWs | 3-day training on CHW core competencies, study design, recruitment strategies and role play/ practice, site visit and discussion of local context. | 6 months | - Diagnosed diabetes and hypertension or an uncontrolled BP reading in the past six months. | No contact with CHWs. | - Received five 60-minutes group health education sessions monthly by CHWs. - Received monthly follow up on action plan through one-on-one and progress note phone calls.   *Theory: Health Belief Model and Social Support Theory* | Not specified | Baseline & 6-month follow-up | - Primary outcome: change in BP control - Secondary outcomes: SBP, DBP, HbA1c, weight, BMI - Other outcomes: physical activity, daily diet intake, medication adherence, diabetes self-management, diabetes physician management, health self-efficacy, depression risk, and days of poor physical and mental health. | - No significant difference in BP control between groups. - SBP, DBP, weight & BMI improved significantly in intervention group. - Greater improvement in medication adherence, engagement in moderate intensity physical activity, and diet control in intervention group. |

*= Pilot randomised controlled trial; BMI= Body mass index; BP= Blood pressure; CHW= Community health worker; CVD= Cardiovascular disease; DBP= Diastolic blood pressure; DSG= Dietitian support group; FBG= Fasting blood glucose; FCHV= Female community health volunteer; HbA1c= glycated haemoglobin; HDL-c= High-density lipoprotein cholesterol; HC= Hip circumference; IFG= Impaired fasting glycaemia; IGT= Impaired glucose tolerance; IRR= Incidence rate ratio; LDL-c= Low-density lipoprotein cholesterol; LHT= Lay health trainer; LSM= Lifestyle modification; MD= Mediterranean diet; MDS= Mediterranean diet score; MSG= Minimal support group; PSG= Peer support group; QOL= Quality of life; SBP= Systolic blood pressure; SD= Standard deviation; T2DM= Type 2 diabetes mellitus; TC= Total cholesterol; TG= Triglycerides; WC= Waist circumference
