# Supplementary material for: Peer-led lifestyle interventions for the primary prevention of cardiovascular disease in community: a systematic review of randomised controlled trials
Source: BMC Public Health. 2024 Mar 14;24:812. doi: 10.1186/s12889-024-18328-w (PMC10941612; doi:10.1186/s12889-024-18328-w)
Supplement: Supplementary file 3 — Supplementary Material 3 [file 12889_2024_18328_MOESM3_ESM.docx]

**Supplementary Table S2. Database search strategy.**

| **OVID MEDLINE** | | |
| --- | --- | --- |
| **#** | **Searches** | **Results** |
| 1 | Cardiovascular Diseases/ or cardiovascular.mp. | 633725 |
| 2 | cardiovascular disease*.mp. | 301985 |
| 3 | cardiac.mp. | 821145 |
| 4 | coronary disease.mp. or Coronary Disease/ | 141237 |
| 5 | CVD.mp. | 41624 |
| 6 | CV.mp. | 54118 |
| 7 | framingham.mp. | 9664 |
| 8 | heart disease*.mp. | 258986 |
| 9 | metabolic syndrome.mp. or Metabolic Syndrome/ | 61564 |
| 10 | 1 or 2 or 3 or 4 or 5 or 6 or 7 or 8 or 9 | 1624847 |
| 11 | peer*.mp. or Peer Group/ | 169435 |
| 12 | peer support.mp. or Social Support/ | 83959 |
| 13 | lay support.mp. | 58 |
| 14 | peer coach.mp. | 54 |
| 15 | peer educator.mp. | 210 |
| 16 | peer counsel*.mp. | 588 |
| 17 | peer led.mp. | 1312 |
| 18 | promotora.mp. | 297 |
| 19 | community health worker.mp. | 1681 |
| 20 | 11 or 12 or 13 or 14 or 15 or 16 or 17 or 18 or 19 | 205022 |
| 21 | lifestyle.mp. | 109552 |
| 22 | behavio*.mp. | 1728132 |
| 23 | nutrition.mp. | 256122 |
| 24 | Diet/ or diet.mp. | 513441 |
| 25 | physical activit*.mp. | 134545 |
| 26 | smoking.mp. or Smoking/ | 304623 |
| 27 | Tobacco/ or tobacco.mp. | 125056 |
| 28 | Exercise/ or exercise.mp. | 392749 |
| 29 | Sleep/ or sleep.mp. | 220415 |
| 30 | 21 or 22 or 23 or 24 or 25 or 26 or 27 or 28 or 29 | 3157963 |
| 31 | 10 and 20 and 30 | 2593 |
| 32 | limit 31 to (english language and yr=“2013-Current” and randomized controlled trial) | 178 |

| **EMBASE** | | |
| --- | --- | --- |
| **#** | **Searches** | **Results** |
| 1 | Cardiovascular Diseases/ or cardiovascular.mp. | 1338689 |
| 2 | cardiovascular disease*.mp. | 545402 |
| 3 | cardiac.mp. | 1252630 |
| 4 | coronary disease.mp. or Coronary Disease/ | 119633 |
| 5 | CVD.mp. | 82433 |
| 6 | CV.mp. | 115784 |
| 7 | framingham.mp. | 19562 |
| 8 | heart disease*.mp. | 526632 |
| 9 | metabolic syndrome.mp. or Metabolic Syndrome/ | 128744 |
| 10 | 1 or 2 or 3 or 4 or 5 or 6 or 7 or 8 or 9 | 2793108 |
| 11 | peer*.mp. or Peer Group/ | 204264 |
| 12 | peer support.mp. or Social Support/ | 126909 |
| 13 | lay support.mp. | 75 |
| 14 | peer coach.mp. | 83 |
| 15 | peer educator.mp. | 327 |
| 16 | peer counsel*.mp. | 1299 |
| 17 | peer led.mp. | 2123 |
| 18 | promotora.mp. | 353 |
| 19 | community health worker.mp. | 2366 |
| 20 | 11 or 12 or 13 or 14 or 15 or 16 or 17 or 18 or 19 | 317327 |
| 21 | lifestyle.mp. | 283744 |
| 22 | behavio*.mp. | 2568853 |
| 23 | nutrition.mp. | 427595 |
| 24 | Diet/ or diet.mp. | 936883 |
| 25 | physical activit*.mp. | 305173 |
| 26 | smoking.mp. or Smoking/ | 622561 |
| 27 | Tobacco/ or tobacco.mp. | 196894 |
| 28 | Exercise/ or exercise.mp. | 658772 |
| 29 | Sleep/ or sleep.mp. | 432902 |
| 30 | 21 or 22 or 23 or 24 or 25 or 26 or 27 or 28 or 29 | 5250786 |
| 31 | 10 and 20 and 30 | 6487 |
| 32 | limit 31 to (english language and yr=“2013-Current” and randomized controlled trial) | 507 |

| **COCHRANE CENTRE FOR CONTROLLED TRIALS** | | |
| --- | --- | --- |
| **#** | **Searches** | **Results** |
| 1 | Cardiovascular Diseases/ or cardiovascular.mp. | 91477 |
| 2 | cardiovascular disease*.mp. | 38275 |
| 3 | cardiac.mp. | 78261 |
| 4 | coronary disease.mp. or Coronary Disease/ | 11154 |
| 5 | CVD.mp. | 7001 |
| 6 | CV.mp. | 7700 |
| 7 | framingham.mp. | 1197 |
| 8 | heart disease*.mp. | 26551 |
| 9 | metabolic syndrome.mp. or Metabolic Syndrome/ | 8803 |
| 10 | 1 or 2 or 3 or 4 or 5 or 6 or 7 or 8 or 9 | 179736 |
| 11 | peer*.mp. or Peer Group/ | 15966 |
| 12 | peer support.mp. or Social Support/ | 5716 |
| 13 | lay support.mp. | 13 |
| 14 | peer coach.mp. | 49 |
| 15 | peer educator.mp. | 109 |
| 16 | peer counsel*.mp. | 343 |
| 17 | peer led.mp. | 790 |
| 18 | promotora.mp. | 101 |
| 19 | community health worker.mp. | 744 |
| 20 | 11 or 12 or 13 or 14 or 15 or 16 or 17 or 18 or 19 | 20418 |
| 21 | lifestyle.mp. | 27294 |
| 22 | behavio*.mp. | 156654 |
| 23 | nutrition.mp. | 32520 |
| 24 | Diet/ or diet.mp. | 77875 |
| 25 | physical activit*.mp. | 44918 |
| 26 | smoking.mp. or Smoking/ | 39097 |
| 27 | Tobacco/ or tobacco.mp. | 11717 |
| 28 | Exercise/ or exercise.mp. | 130164 |
| 29 | Sleep/ or sleep.mp. | 51649 |
| 30 | 21 or 22 or 23 or 24 or 25 or 26 or 27 or 28 or 29 | 420808 |
| 31 | 10 and 20 and 30 | 867 |
| 32 | limit 31 to (english language and yr=“2013-Current”) | 680 |

| **PUBMED** | | |
| --- | --- | --- |
| **#** | **Searches** | **Results** |
| 1 | ((((((((cardiovascular) OR (cardiovascular disease*)) OR (heart disease)) OR (cardiac)) OR (coronary disease)) OR (cvd)) OR (cv)) OR (metabolic syndrome)) OR (framingham) | 3,664,601 |
| 2 | ((((((((((peer*) OR (peer support)) OR (social support)) OR (lay support)) OR (peer coach)) OR (peer educator)) OR (peer counsel*)) OR (peer led)) OR (peer group)) OR (promotora) OR (community health worker) | 753,289 |
| 3 | ((((((((lifestyle) OR (behavio*)) OR (nutrition)) OR (diet)) OR (physical activit*)) OR (smoking)) OR (tobacco)) OR (exercise)) OR (sleep) | 4,672,069 |
| 4 | ((((((((((cardiovascular) OR (cardiovascular disease*)) OR (heart disease)) OR (cardiac)) OR (coronary disease)) OR (cvd)) OR (cv)) OR (metabolic syndrome)) OR (framingham) AND ((((((((((peer*) OR (peer support)) OR (social support)) OR (lay support)) OR (peer coach)) OR (peer educator)) OR (peer counsel*)) OR (peer led)) OR (peer group)) OR (promotora)) OR (community health worker))) AND (((((((((lifestyle) OR (behavio*)) OR (nutrition)) OR (diet)) OR (physical activit*)) OR (smoking)) OR (tobacco)) OR (exercise)) OR (sleep)) | 21,341 |
| 5 | ((((((((((cardiovascular) OR (cardiovascular disease*)) OR (heart disease)) OR (cardiac)) OR (coronary disease)) OR (cvd)) OR (cv)) OR (metabolic syndrome)) OR (framingham) AND ((((((((((peer*) OR (peer support)) OR (social support)) OR (lay support)) OR (peer coach)) OR (peer educator)) OR (peer counsel*)) OR (peer led)) OR (peer group)) OR (promotora)) OR (community health worker))) AND (((((((((lifestyle) OR (behavio*)) OR (nutrition)) OR (diet)) OR (physical activit*)) OR (smoking)) OR (tobacco)) OR (exercise)) OR (sleep)) Filters: in the last 10 years | 14,055 |
| 6 | ((((((((((cardiovascular) OR (cardiovascular disease*)) OR (heart disease)) OR (cardiac)) OR (coronary disease)) OR (cvd)) OR (cv)) OR (metabolic syndrome)) OR (framingham) AND ((((((((((peer*) OR (peer support)) OR (social support)) OR (lay support)) OR (peer coach)) OR (peer educator)) OR (peer counsel*)) OR (peer led)) OR (peer group)) OR (promotora)) OR (community health worker))) AND (((((((((lifestyle) OR (behavio*)) OR (nutrition)) OR (diet)) OR (physical activit*)) OR (smoking)) OR (tobacco)) OR (exercise)) OR (sleep)) Filters: in the last 10 years, English | 13,961 |
| 7 | ((((((((((cardiovascular) OR (cardiovascular disease*)) OR (heart disease)) OR (cardiac)) OR (coronary disease)) OR (cvd)) OR (cv)) OR (metabolic syndrome)) OR (framingham) AND ((((((((((peer*) OR (peer support)) OR (social support)) OR (lay support)) OR (peer coach)) OR (peer educator)) OR (peer counsel*)) OR (peer led)) OR (peer group)) OR (promotora)) OR (community health worker))) AND (((((((((lifestyle) OR (behavio*)) OR (nutrition)) OR (diet)) OR (physical activit*)) OR (smoking)) OR (tobacco)) OR (exercise)) OR (sleep)) Filters: Randomized Controlled Trial, in the last 10 years, English | 1,082 |

| **SCOPUS** | | |
| --- | --- | --- |
| **#** | **Searches** | **Results** |
| 1 | Cardiovascular OR Cardiovascular disease* OR Heart disease OR Cardiac OR Coronary disease OR CVD OR CV OR Metabolic syndrome OR Framingham AND Peer* OR Peer support OR Social support OR Lay support OR Peer coach OR Peer educator OR Peer counsel* OR Peer led OR Peer group OR Promotora OR Community health worker AND Lifestyle OR Behavio* OR Nutrition OR Diet OR Physical activit* OR Smoking OR Tobacco OR Exercise OR Sleep AND PUBYEAR > 2012 AND PUBYEAR < 2024 AND ( LIMIT-TO ( LANGUAGE,"English" ) ) | 5,563 |
| 2 | Cardiovascular OR Cardiovascular disease* OR Heart disease OR Cardiac OR Coronary disease OR CVD OR CV OR Metabolic syndrome OR Framingham AND Peer* OR Peer support OR Social support OR Lay support OR Peer coach OR Peer educator OR Peer counsel* OR Peer led OR Peer group OR Promotora OR Community health worker AND Lifestyle OR Behavio* OR Nutrition OR Diet OR Physical activit* OR Smoking OR Tobacco OR Exercise OR Sleep AND PUBYEAR > 2012 AND PUBYEAR < 2024 AND ( LIMIT-TO ( LANGUAGE,"English" ) ) AND ( EXCLUDE ( EXACTKEYWORD,"Animals" ) OR EXCLUDE ( EXACTKEYWORD,"Animal" ) ) AND ( LIMIT-TO ( EXACTKEYWORD,"Controlled Study" ) ) | 689 |
